# Switchable sensitizers stepwise lighting up lanthanide emissions

**DOI:** 10.1038/srep09335

**Published:** 2015-03-20

**Authors:** Yan Zhang, Peng-Chong Jiao, Hai-Bing Xu, Ming-Jing Tang, Xiao-Ping Yang, Shaoming Huang, Jian-Guo Deng

**Affiliations:** 1 New Materials R&D Center, Institute of Chemical Materials, China Academy of Engineering Physics, Mianyang, Sichuan, 621900, China; 2 Key Laboratory of Science and Technology on High Energy Laser, Si Chuan Research Center of New Materials, Chengdu, Sichuan 610207, China; 3 College of Chemistry and Materials Engineering, WenZhou University, Wenzhou, Zhejiang 325035, China

## Abstract

Analagous to a long-ranged rocket equipped with multi-stage engines, a luminescent compound with consistent emission signals across a large range of concentrations from two stages of sensitizers can be designed. In this approach, ACQ, aggregation-caused quenching effect of sensitizers, would stimulate lanthanide emission below 10^−4^ M, and then at concentrations higher than 10^−3^ M, the “aggregation-induced emission” (AIE) effect of luminophores would be activated with the next set of sensitizers for lanthanide emission. Simultaneously, the concentration of the molecules could be monitored digitally by the maximal excitation wavelengths, due to the good linear relationship between the maximal excitation wavelengths and the concentrations {lg(*M*)}. This model, wherein molecules are assembled with two stages (both AIE and ACQ effect) of sensitizers, may provide a practicable strategy for design and construction of smart lanthanide bioprobes, which are suitable in complicated bioassay systems in which concentration is variable.

Single-molecule fluorescence techniques are crucial for numerous applications, such as cell biology and early diagnosis^1^,^2^,^3^. With the development of bioprobes, ratiometric probes with high sensitivity to the changes in the concentration of a range of analytes have been exploited^4^,^5^. However, the ubiquitous “aggregation-caused quenching” (ACQ) effect has hampered the detection of individual fluorescent molecules in solution at high concentrations^6^,^7^,^8^. Ideally, it is desirable that the output signals from bioprobes in any concentrations possess high sensitivity and resolution so that consistent signals from the bioprobes could still be obtained in complicated bioassay systems, as the bioprobes may accumulate on the surfaces of the biomacromolecules with different concentrations^9^.

Inspired by the fact that multistage propulsion systems propel a rocket stepwise across a route, consistent lanthanide emissions of high sensitivity and resolution across a wide range of concentrations could likewise be achieved by employing a strategy, in which different kinds of sensitizers are activated stepwise for efficient lanthanide emission at different concentrations.

Recently, a cyclic lanthanide complex with reversible “walkable dual emissions”, whose emission is dependent on concentration and temperature by a vibronic mechanism, has been demonstrated by our group^10^. This technique is a promising concept model for signal exchanging and dispatching. Herein, a new type of luminescent lanthanide complex [TPE-TPY-Eu(hfac)_3_] (**1**, hfac^−^ = hexafluoroacetylacetonate; TPE-TPY = 4′-(4-(1,2,2-triphenylvinyl)phenyl)-2,2′:6′,2′′-terpyridine) is reported (Scheme 1 and Figure S1(SI)). It is equipped with two types of chromophores: one is TPE-TPY (^1^H NMR and ^13^C NMR (Figures S2–S3, SI) which has a “bladed” structure with multiple aryl groups and can provide “aggregation-induced emission” (AIE) effect^11^; the other one is a planar luminogen^12^ of hfac^−^ which has the “aggregation-caused quenching” (ACQ) effect at high concentrations^6^. Photophysical behaviour show that hfac^−^ mainly acts as the sensitizer to cause the Eu^III^-based emission at a very low concentration (≤10^−5^ M). With the concentration climbing to the 10^−4^ M to 10^−3^ M range, both hfac^−^ and TPE-TPY pump their energies into the Eu^III^ excited states. Once the concentration surpasses 10^−3^ M, TPE-TPY is activated as the sole energy donor by aggregation effect. It is believed that this is the first example in which different types of chromophores are stepwise-activated to act as sensitizers for efficient lanthanide emission with the concentrations of the complex as the variable.

## Results

### Energy transfer procedures

**Figure 1:**
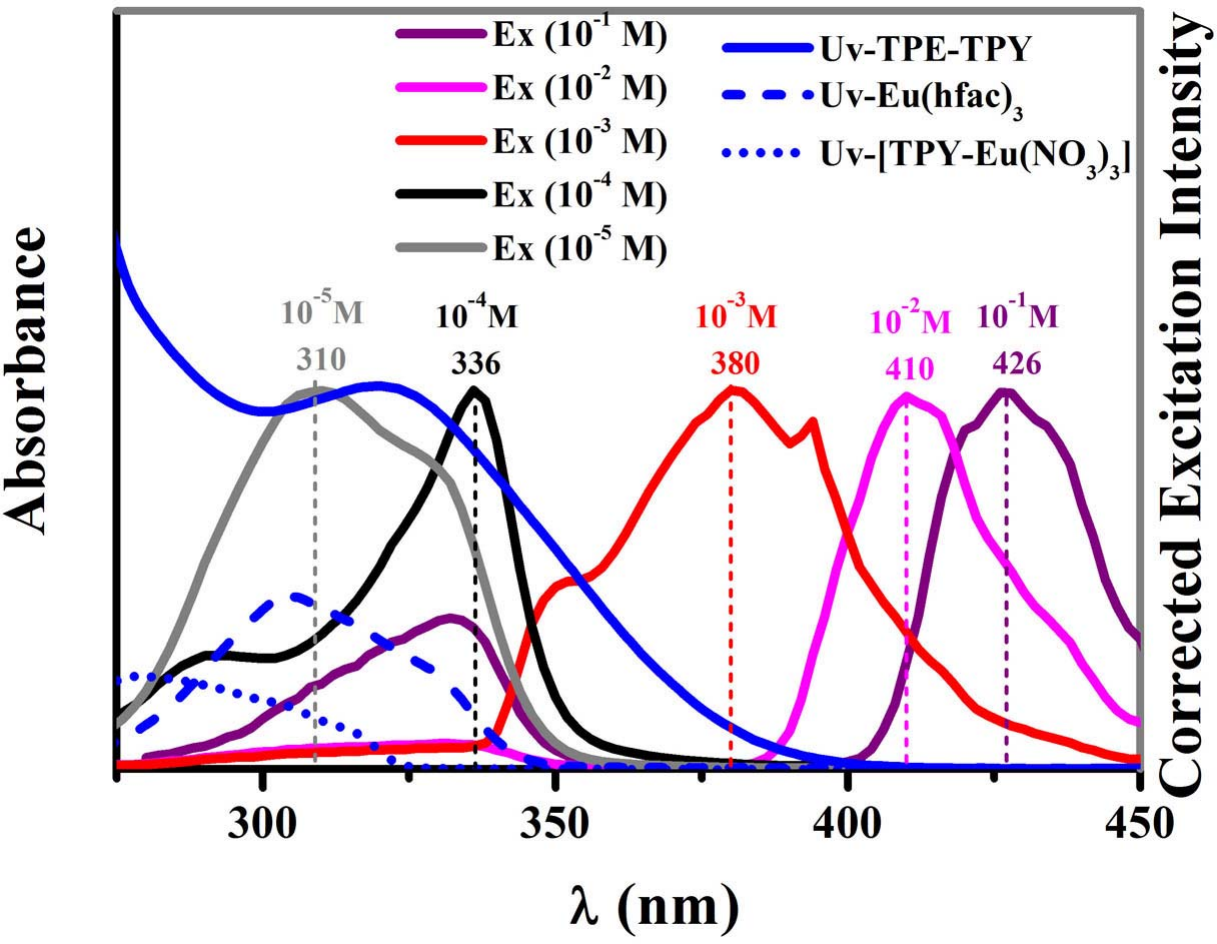
Excitation spectra of 1 (*λ*_em_ = 613 nm) in different concentrations, and absorption spectra of TPE-TPY, Eu(hfac)_3_·2H_2_O and [TPY-Eu(hfac)_3_] in CH_2_Cl_2_ with the concentrations of 10^−5^ M at 298 K.

The photophysical properties of 1, as well as related complexes [TPE-TPY-Gd(hfac)_3_], TPE-TPY, Eu(hfac)_3_·2H_2_O, [TPY-Eu(NO_3_)_3_] and [TPE-TPY-Eu(NO_3_)_3_], were studied. The reference [TPE-TPY-Gd(hfac)_3_], which lacks energy transfer from the TPE-TPY antenna triplet states, exhibits ligand phosphorescence as two vibronic shoulders at *λ*_max_ = 452 and 475 nm, corresponding to the vibrational stretching frequency of the pyridyl groups (Figure S4, SI)^14^. The calculated triplet-state energy level (21100 cm^−1^) is favourable for energy transfer into that of Eu^3+^ (^5^D_0_, 17300 cm^−1^)^15^. However, the excitation spectrum of 1 (Figure 1, *λ*_max_ = 310 nm, gray line) with a concentration of 10^−5^ M only resembles the profile of the absorption spectrum of the precursor Eu(hfac)_3_·2H_2_O rather than that of [TPY-Eu(NO_3_)_3_], indicating an energy transfer pathway is mainly from the hfac^−^ instead of TPY to the Eu^3+^ ion (the quantum efficiency (*η*) is ca. 42.5%) in very dilute solutions (≤10^−5^ M). Additionally, the emission intensity (*λ*_ex_ = 306 nm) of [TPE-TPY-Eu(NO_3_)_3_] is very weak and much lower than that of [TPY-Eu(NO_3_)_3_] (Figure S5, SI). These facts indicate that the intramolecular rotations of TPE consume the energies of the excited states of TPY, markedly reducing the efficiency of the energy transfer from TPY to Eu^III^ ion. Consequently, hfac^−^ acts as the main sensitizer for Eu^III^-based emission in 1 at low concentration.

When the concentration climbs up to 10^−4^ M, the excitation spectrum of **1** is a hybrid of the profiles of the absorption spectra of Eu(hfac)_3_·2H_2_O and TPE-TPY, and the excitation window red shifts from 310 nm to 336 nm (Figure 1, black line). These facts indicate that hfac^−^ and TPE-TPY are all activated as the sensitizers for the Eu^III^-based emission. When the concentration is higher than 10^−3^ M, since only the absorption spectrum of TPE-TPY appears within the regions of the excitation window (Figure 1, *λ*_max_ = 380 nm, red line), the TPE-TPY solely contributes to the Eu^III^-based emission. This conclusion also can be verified by two facts: i) Eu(hfac)_3_·2H_2_O^16^ or [TPY-Eu(NO_3_)_3_] without TPE does not show lanthanide luminescence at excitation wavelength *λ*_ex_ > 350 nm (Figure 1); ii) a related compound [TPE-TPY-Eu(NO_3_)_3_] without hfac^−^ exhibits a typical Eu^III^-based emission (Figure S6, SI) at higher concentration (10^−3^ M) with *λ*_ex_ = 380 nm, while no such emission is found at lower concentration (10^−5^ M).

### Photophysical properties

#### Photophysical properties of TPE-TPY

**Figure 2:**
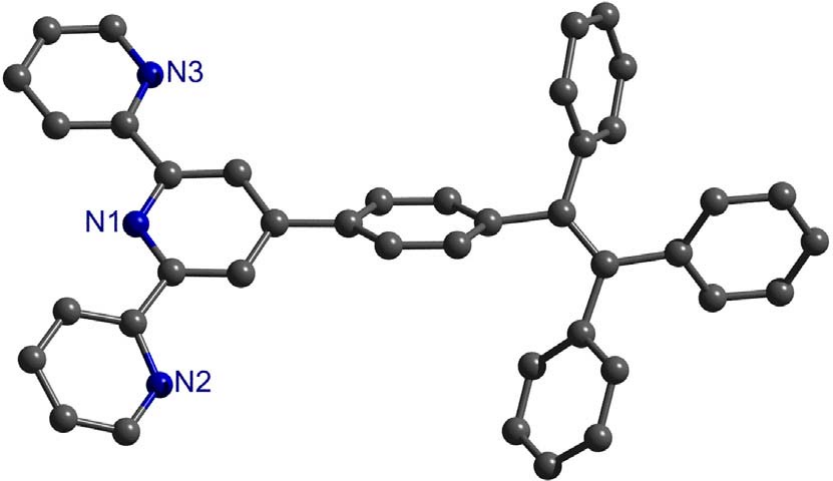
Perspective drawings of TPE-TPY with atom-labeling scheme.

The free TPE-TPY can show obvious AIE phenomenon in different concentrations. As shown in Figure 2, the pyridyl and phenyl rings in free TPE-TPY are not coplanar; instead, they are twisted with respect to each other, and consequently they induce rapid intramolecular rotations in an isolated state. Since the intramolecular rotations effectively consume the energies of the excited states^11^, TPE-TPY is almost non-emissive in dilute solutions (Figure S7, SI). In high concentrations such as more than 10^−3^ M, owing to the rigid restriction on the molecular rotations in aggregate state, the locations of phenyl rings in TPE-TPY are locked, leading to a restriction of intramolecular rotation (RIR) process^11^ therefore making TPE-TPY highly emissive (Figure S7, SI).

As a typical AIE compound, the free TPE-TPY has the following interesting photophysical behaviours. (1) With increasing concentration, the emission intensities are progressively enhanced, and the central emission bands experience little red shifts (10 nm). When the concentration reaches its highest value in the solid state, the emission intensities climb markedly to the highest one, and the area of the emission profiles is 3500 times larger than that of the lowest one with the concentration of 10^−5^ M in dichloromethane solutions (Figure S7, SI). (2) The addition of large amounts of *n*-hexane solvent (a poor solvent of TPE-TPY), which causes the molecules of TPE-TPY to aggregate, can enhance their emission performances (Figure S8, SI). Once the *n*-hexane contents in the solvent mixtures (dichloromethane/*n*-hexane) exceed 50%, the emission progressively intensifies as the *n*-hexane content increased. Meanwhile, the emission spectra exhibit slightly bathochromic-shifts^17^.

### AIE phenomena of 1

**Figure 3:**
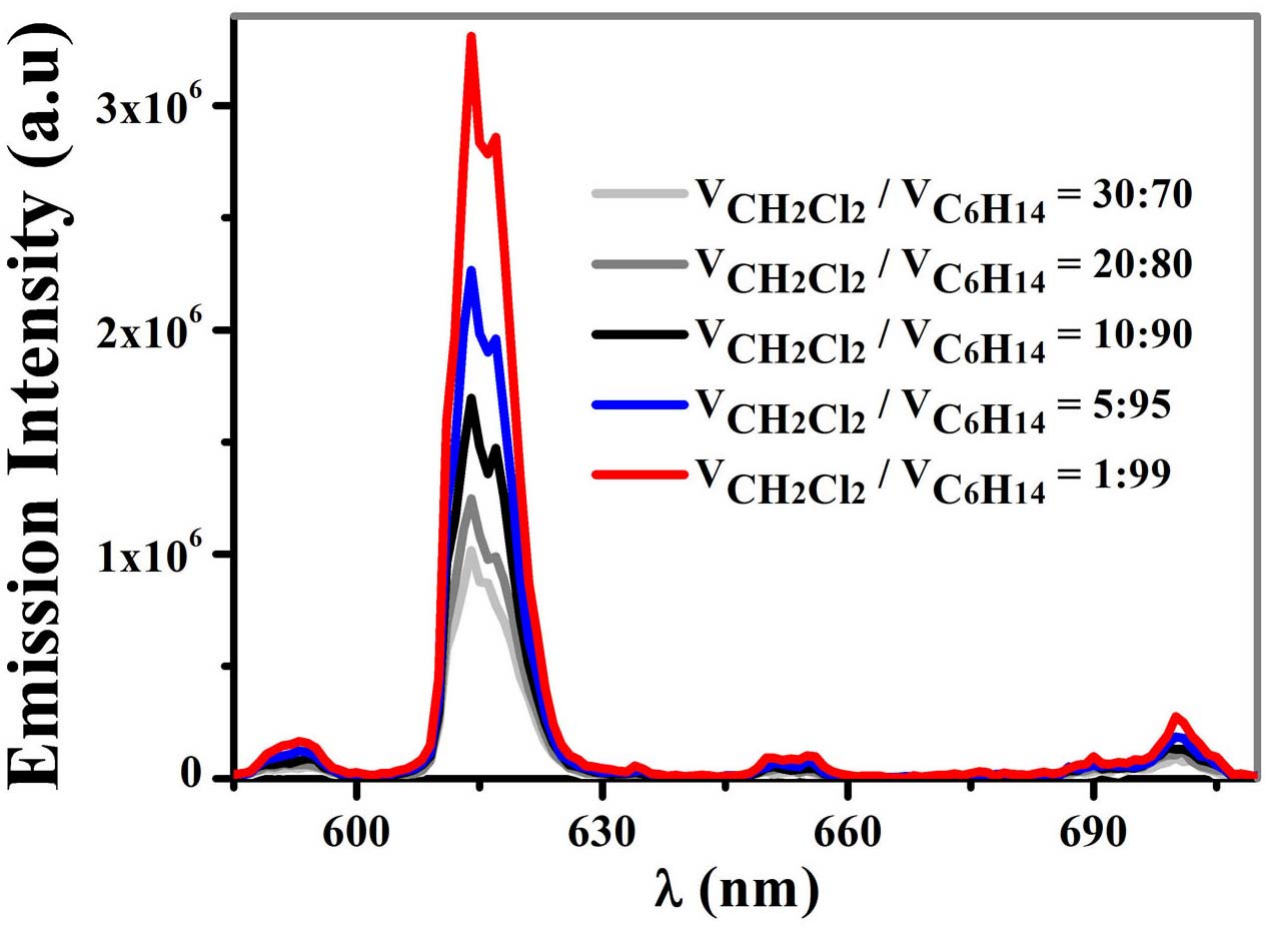
Emission spectra (*λ*_ex_ = 336 nm) of 1 (1 × 10^−4^ M) in dichloromethane/*n*-hexane mixtures containing different volume fractions of *n*-hexane.

In our case, as shown in Figure 3, when the initial concentration of **1** is more than 10^−4^ M, the Eu^III^-based emission is largely improved by increasing the poor solvent *n*-hexane contents in the mixed solvents (starting from 70% content of *n*-hexane). This may be due to the fact that the addition of *n*-hexane causes the aggregation of **1**, resulting in the formation of nanoparticles in the mixed solvents^17^. With larger size and more population of the nanoparticles of **1**, the Eu^III^-based emission in 99% *n*-hexane is about three times as strong as that in 70% *n*-hexane. It is noticeable that the addition of *n*-hexane results in the enhancement of the absorption intensities of **1** (Figure S9, SI), due to the light scattering of the nanoparticles formed in the mixed solvents^18^.

### Monitored the concentration online

**Figure 4:**
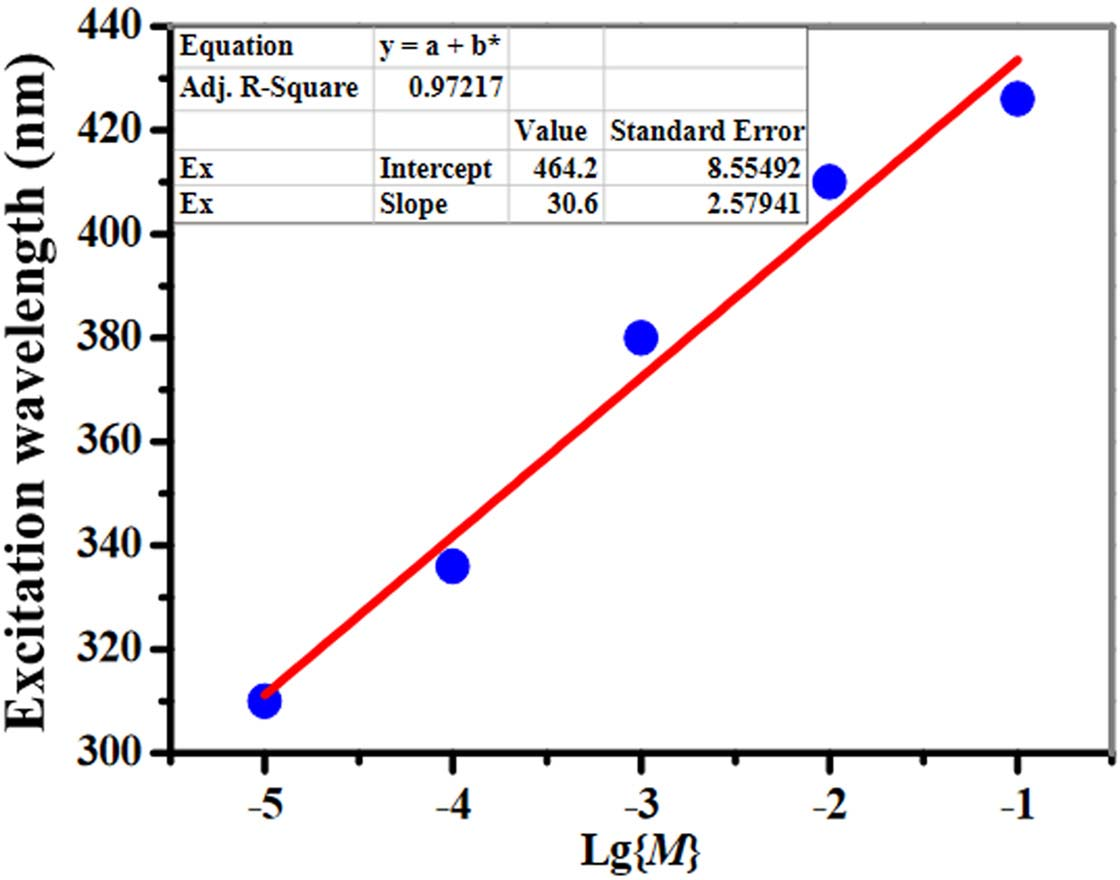
Linear relationships between the maximal excitation wavelength *vs* the concentrations {lg(*M*)} of 1 at 298 K.

The energy transfer procedures could be simply viewed as this: there are two kinds of fuels attached to a rocket. Once the first fuel is about to run out, the second one would immediately work to push the rocket into the sky. As the quantity of the fuel is the key point to start the second one, monitoring the concentration realtime is very important. As shown in Figure 4, different sensitizers are activated for Eu^III^-based emission by increasing the concentrations; the maximal excitation wavelengths extend from ca. 310 nm to 426 nm, indicating that small molecules with variable concentrations could be stimulated to emit by different excitation wavelengths. Surprisingly, there is a good linear relationship between the maximal excitation wavelength and the concentration {lg(*M*)} (Figure 4, *R*^2^ = 0.97) of 1; thus not only high-resolution lanthanide signals could be obtained, but also the varying concentrations of the molecules could be evaluated realtime by the maximal excitation wavelength.

## Discussions

### Tendency of the emission efficiency

**Figure 5:**
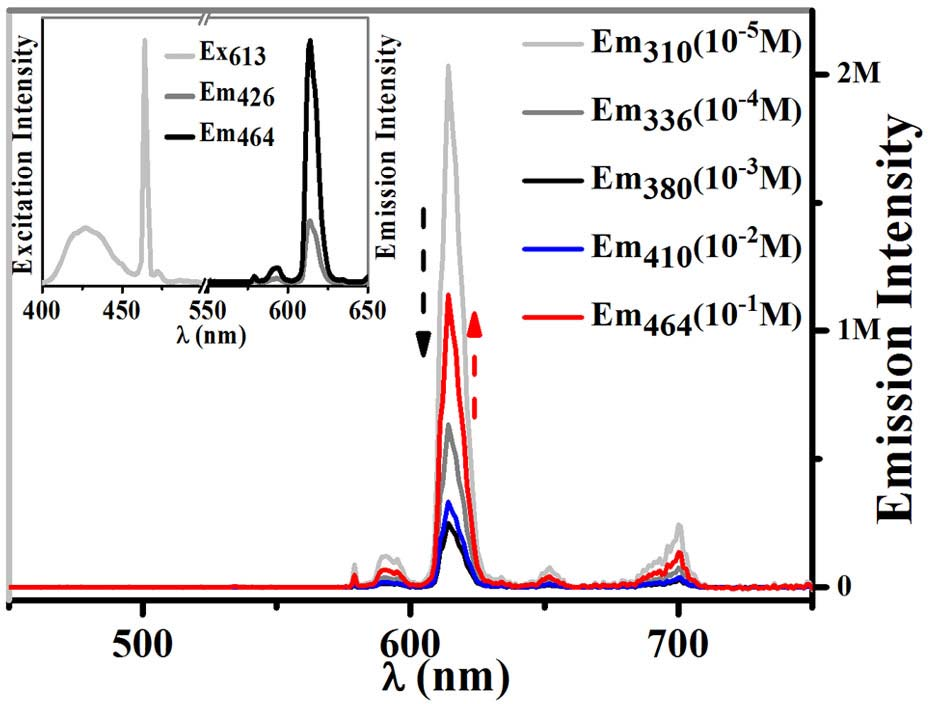
Emission spectra of 1 with different concentrations in CH_2_Cl_2_ at 298 K.

We were naturally interested in the influence of different sensitizers in the Eu^III^-based emission intensities of 1 at different concentrations. It has been demonstrated that *β*-diketonates are one of the best sensitizers for Eu^III^-based emission in dilute solutions^15^,^20^,^25^. With hfac^−^ acting as the main sensitizer for lanthanide emission in dilute solution (10^−5^ M), the quantum yield (*Φ*_em_) of Eu^III^ in 1 is 3.95 ± 0.15% (Figure 5). When the concentration climbs to 10^−4^ M, both hfac^−^ and TPE-TPY are all activated as the sensitizers for the Eu^III^-based emission. However, the Eu^III^-based emission intensity is lower than that at 10^−5^ M, with a quantum yield of 0.645 ± 0.015%. This may be due to the fact that the ACQ effect on hfac^−^ decreases its sensitizer performance and the AIE effect on TPE-TPY is not activated at this concentration (Figure S7, SI). When the concentration exceeds 10^−3^ M, the emission performance of the TPE-TPY is just activated due to the AIE effect (Figure S7, SI), and only TPE-TPY serves as the sensitizer (Figure 1). Consequently the Eu^III^-based emission intensity of 1 becomes the lowest one (*Φ*_em_ = 0.160 ± 0.001% at 10^−3^ M). Once the AIE effect further promotes the emission performance of TPE-TPY, the Eu^III^-based emission intensity of 1 becomes higher and higher with the increase of concentrations (Figure 5). And the quantum yield of Eu^III^ is 0.99 ± 0.04% when the concentration of 1 is 10^−1^ M. So when the concentrations of 1 change from low to high, the emission intensities of Eu^III^ change in the order: high-low-high (Figure 5). In return, upon diluting the solution from high concentration such as 10^−1^ M, the same phenomena will happen due to the non-destructive physical cycles.

### An inferential mechanism of 1

Due to the forbidden Laporte rule and low molar absorption coefficient, the lanthanide ions could only be efficiently populated by adjacent absorbing light harvesting, which act as the sensitizers to pump their energies into the lanthanide excited states by antenna effect^19^. Since the f-f′ transitions in lanthanide ion are inert^15^, the pumped excited energies from the sensitizer could be employed to modulate the performance of the sensitized lanthanide emissions^20^,^21^.

**Figure 6:**
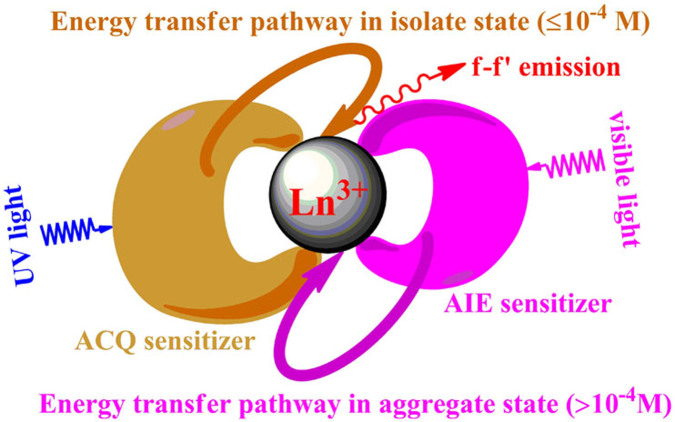
Diagram of different chromophores acting as the alternative sensitizers for lanthanide emission by increasing the concentration.

In this case, as shown in Figure S10 (SI), hfac^−^ mainly acts as the first sensitizer to light up the Eu^III^-based emission at very low concentrations. When the concentrations increase, the ACQ effect on hfac^−^ decreases the sensitizer performance, so as to weaken the intensity of the Eu^III^-based emission. Then the RIR processes is activated on TPE-TPY by aggregation (≥10^−3^ M), switching the energy transfer pathway from TPE-TPY instead of hfac^−^ to the Eu^III^-subunit, thus the AIE effect on TPE-TPY improves the efficiency of the Eu^III^-based emission. Consequently, as depicted in Figure 6, hfac^−^, hfac^−^/TPE-TPY, and TPE-TPY are activated stepwise to act as the dominated sensitizers for the efficient Eu^III^-based emission at different concentrations, moreover the excitation wavelength (Figure 1) and luminescence quantum yields (Figure 5) of Eu^III^ complex can be easily tuned by the ACQ/AIE process of the sensitizers.

Although the AIE has been reported for many years^22^, it is still challenging to achieve the AIE effect on organometallic complexes by the antenna effect^23^. Since the electronic transitions of organometallic luminogens and AIE functional groups originate from the outer shells, their excitation energies are all susceptible to the external stimulations. Interestingly, as shown in above, in **1** the excited energies induced by aggregation in TPE-TPY are efficiently transferred to the Eu^III^-subunit. Thus we firstly accomplish to introduce the AIE in lanthanide complex systems, thanks to the shielding effect and the inner f-f′ electronic transitions in lanthanide(III) ions. Based on the above possible mechanism, asides from acting as bioprobe and sensors, such kind of lanthanide may be promising in other relative applications, such as in solid-state emitters and so on^24^.

In summary, a new type of lanthanide complex **1** equipped with both the AIE and ACQ effects of antennae, which can be stepwise-activated to act as sensitizers for efficient lanthanide emission, has been prepared. Due to “dual sensitization pathways” in **1**, that is using a planar structure of sensitizer with ACQ effect to light up Ln^III^-based emission within low concentrations, then a “bladed” structure of antenna with AIE effect to simulate the lanthanide(III) emission at high concentrations, consistent signals of Eu^III^-based emission across wide range of concentrations are achieved by triggering the ACQ/AIE process. To the best of our knowledge, this is the first work by introducing the AIE in lanthanide complex systems. Regarding the application of the “dual sensitization pathways” concept in the design of lanthanide bioprobes, a smart ionic lanthanide bioprobes (Figure S12, SI) with strong output signals within a large scale of concentrations would be constructed by such strategy.

## Methods

### Sample Preparation

All manipulations were performed under dry argon atmosphere using Schlenk techniques and a vacuum-line system. The solvents were dried, distilled, and degassed prior to use, except those for spectroscopic measurements were of spectroscopic grade. Hexafluoroacetylacetone (Hhfac) was commercially available. Ln(hfac)_3_·2(H_2_O)_2_ (Ln = Eu, Gd)^26^, 4-(1,2,2-triphenylvinyl)phenyl)boronic acid (TPE-B(OH)_2_)^13^, and 4′-chloro-2,2′:6′,2″-terpyridine (Cl-TPY)^27^ were prepared by the literature procedures.

### Synthesis of 4′-(4-(1,2,2-triphenylvinyl)phenyl)-2,2:6′,2″-terpyridine (TPE-TPY)

A mixture of TPE-B(OH)_2_ (0.564 g, 1.5 mmol), Cl-TPY (0.267 g, 1 mmol), Pd(PPh_3_)_4_ (0.202 g, 0.2 mmol), and K_2_CO_3_ (2.8 g, 20 mmol) was dissolved in degassed toluene/ethanol/water (120 mL, V:V:V = 8:2:2) and then refluxed for 24 h under argon atmosphere. The solution was cooled to room temperature and washed with brine (100 mL). The organic layer was dried over magnesium sulfate, filtered, and concentrated to afford the crude product. The crude product was purified by column chromatography on silica to afford white power (Yield 40%). ^1^H NMR (400 MHz, CDCl_3_): *δ* (ppm):8.70–8.82 (m, 3H), 7.97 (d, *J* = 7.8 Hz, 2H), 7.66 (d, *J* = 8.0 Hz, 1H), 7.47 (d, *J* = 8.4 Hz, 2H), 6.93–7.10 (m, 21H). ^13^C NMR (100 MHz, CDCl_3_) *δ* (ppm):155.0, 149.4, 147.4, 146.6, 143.4, 143.3, 142.1, 140.3, 139.9, 135.6, 135.0, 133.1, 132.3, 132.2, 132.0, 131.9, 131.4, 128.6, 127.9, 127.8, 127.7, 126.8, 126.7, 126.3, 123.9, 120.1, 117.7, 115.9, 114.5.

### Synthesis of [TPE-TPY-Ln(hfac)_3_] (Ln = Eu, Gd)

Ln(hfac)_3_·2(H_2_O)_2_ (Ln = Eu, Gd) (0.1 mmol) and equal molar ratio (one equivalent) of TPE-TPY were stirred in 30 mL dichloromethane at ambient atmosphere until the solution became clear. After filtration, crystallization by layering *n*-hexane onto the corresponding concentrated dichloromethane solutions afforded the products as crystals.

### 1

Anal. Calcd for C_56_H_32_N_3_EuF_18_O_6_: C, 50.31; H, 2.41; N, 3.14. Found: C, 50.44; H, 2.41; N, 3.15. IR (KBr, cm^−1^): 1650 s (C = O), 1256 s (C = C/C-CF_3_). ESI-MS (CH_3_OH-CH_2_Cl_2_, *m/z*): 1338 [M + H]^+^ (Figure S13, SI). Yield: 98%.

### [TPE-TPY-Gd(hfac)_3_]

Anal. Calcd for C_56_H_32_N_3_GdF_18_O_6_: C, 50.12; H, 2.40; N, 3.13. Found: C, 50.14; H, 2.42; N, 3.14. IR (KBr, cm^−1^): 1651 s (C = O). Yield: 97%.

### Synthesis of [TPE-TPY-Eu(NO_3_)_3_]

This compound was prepared by the same procedure as that of [TPE-TPY-Ln(hfac)_3_] except for using Eu(NO_3_)_3_(H_2_O)_2_ instead of Eu(hfac)_3_(H_2_O)_2_ to give the product as white crystals. Yield: 97%. Anal. Calcd for C_41_H_29_N_6_EuO_9_: C, 54.62; H, 3.24; N, 9.32. Found: C, 54.64; H, 3.21; N, 9.35.

### Synthesis of [TPY-Eu(NO_3_)_3_]

This compound was prepared by the same procedure as that of [TPE-TPY-Eu(NO_3_)_3_] except for using TPY instead of TPE-TPY to give the product as white crystals. Yield: 98%. Anal. Calcd for C_15_H_11_N_6_EuO_9_: C, 31.54; H, 1.94; N, 14.71. Found: C, 31.56; H, 1.93; N, 14.68.

### Physical Measurements

Elemental analyses (C, H, N) were carried out on a Perkin-Elmer model 240C elemental analyzer. Electrospray ion mass spectra (ESI−MS) were performed on a Finnigan LCQ mass spectrometer using dichloromethane-methanol mixture as mobile phases. UV-vis absorption spectra were measured on a Perkin-Elmer Lambda 35 UV-vis spectrophotometer. Infrared (IR) spectra were recorded on a Magna750 FT-IR spectrophotometer with KBr pellet. Emission, excitation spectra and emission lifetimes were recorded on an Edinburgh Instrument (FLS 920 spectrometer, the ratio between signal to noise ca. 6000:1 by using the Roman peak of water) with the same slit (1.9980 mm) and iris (10, the largest one is 100) in our experiments. The emission spectra (Figure S6, SI) for concentrations less than 10^−5^ M are not operative, due to instrument limitation. The quantum yields of 1 with different concentrations in degassed dichloromethane were determined relative to that of [Ru(bpy)_3_]Cl_2_ (*Φ*_em_ = 0.028) in H_2_O^28^,^29^. All the quantum yields were calculated by *Φ*_s_ = *Φ*_r_(*A*_r_/*A*_s_)(*I*_r_/*I*_s_)(*n*_s_/*n*_r_)^2^(*D*_s_/*D*_r_)^28^, where the subscript r and s denote reference standard and the sample solution, respectively; and *A*, *n*, *I*, *D* and *Φ* are the absorbance of a sample at excitation wavelength *λ*, the refractive index of the solvents, the relative intensity of excitation light at wavelength *λ*, the integrated intensity and the luminescence quantum yield, respectively. All the solutions used for the determination of emission lifetimes and quantum yields were prepared under vacuum in a 10 cm^3^ round bottom flask equipped with a side arm 1 cm fluorescence cuvette and sealed from the atmosphere by a quick-release Teflon stopper. Solutions used for luminescence determination were prepared after rigorous removal of oxygen by three successive freeze-pump-thaw cycles. For example, [Ru(bpy)_3_]Cl_2_ in H_2_O and TPE-TPY-Eu(hfac)_3_ in CH_2_Cl_2_ possess approximate absorption coefficient within 0.05 at 310 nm with the concentration of TPE-TPY-Eu(hfac)_3_ at 10^−5^ M; Similarly, they have approximate absorption coefficient within 0.05 at 336 nm with the concentration of TPE-TPY-Eu(hfac)_3_ at 10^−4^ M, and 380 nm at 10^−3^ M. Meantime, the slit (1.988 mm) and iris (10) are the same for excitation and emission operation for all compounds in measurements.

The quantum efficiency is calculated from the equation *η* = [*A*_rad_/(*A*_rad_ + *A*_nrad_)], in which, *A*_rad_ may be determined from the emission spectra by the usual equation *A*_rad_ = *A*_MD,0_ × *n*^3^ × *I*_total_/*I*_MD_, where *A*_MD,0_ is the probability of spontaneous emission for the ^5^D_0_ → ^7^F_1_ transition in vacuum (14.65 s^−1^); *n* is the refractive index of the medium; and *I*_total_/*I*_MD_ is the ratio of the integrated area of the intensities of the emission spectrum with respect to the integrated area of the magnetic dipole transition ^5^D_0_ → ^7^F_1_. On the other hand, *A*_rad_ + *A*_nrad_ is equal to 1/*τ*_obs_ where *τ*_obs_ can be determined from an exponential fitting of the lifetime decay curve^30^.

### Crystal Structural Determination

Single crystal of TPE-TPY (CCDC 958536) suitable for *X*-ray diffraction was grown by layering *n*-hexane onto the corresponding dichloromethane solutions. Crystals coated with epoxy resin or sealed in capillaries with mother liquors were measured on a SIEMENS SMART CCD diffractometer by *ω* scan technique at room temperature using graphite-monochromated Mo-K*α* radiation (*λ* = 0.71073 Å). Lp corrections were carried out in the reflection reduction process. The structures were solved by direct method. The remaining non-hydrogen atoms were determined from the successive difference Fourier syntheses. The non-hydrogen atoms were refined anisotropically except for the F atoms, and the hydrogen atoms were generated geometrically with isotropic thermal parameters. The structures were refined on *F*^2^ by full-matrix least-squares methods using the SHELXTL-97 program package. Crystallographic data of TPE-TPY was summarized in Table S1.

## Author Contributions

H.X. and Y.Z. carried out the experimental work. H.X. contributed to the design of the experiments and finished the writing of the paper. H.X., Y.Z., P.J., M.T. and S.H. contributed to the analysis of the data. X.Y. and J.D. gave advices on the writing of the manuscripts. All the authors reviewed the paper.

## Supplementary Material

Supplementary Information Additional experimental and spectroscopic data together with X-ray crystallographic files of compound TPE-TPY.
