# Supplementary material for: Switchable sensitizers stepwise lighting up lanthanide emissions
Source: Sci Rep. 2015 Mar 20;5:9335. doi: 10.1038/srep09335 (PMC4366845; doi:10.1038/srep09335)
Supplement: Supplementary Information — Additional experimental and spectroscopic data together with X-ray crystallographic files of compound TPE-TPY. [file srep09335-s1.doc]

**Supplementary Information**

***Switchable sensitizers stepwise lighting up lanthanide emissions***

Yan Zhang,*^a,b^* Peng-Chong Jiao,*^a,b^* Hai-Bing Xu,**^a,b^* Ming-Jing Tang,*^a,b^* Xiao-Ping Yang,*^c^* Shaoming Huang,*^c^* and Jian-Guo Deng**^a,b^*

^a^New Materials R&D Center, Institute of Chemical Materials, China Academy of Engineering Physics, Mianyang, Sichuan, 621900, China.

^b^Key Laboratory of Science and Technology on High Energy Laser, Si Chuan Research Center of New Materials, Chengdu, Sichuan 610207, China. *Corresponding Author: E-mail:hai_bingxu@163.com; d13258430956@126.com; Fax: (+) 86-28-8588-0792.

^c^*College of Chemistry and Materials Engineering, WenZhou University, Wenzhou, Zhejiang 325035*, *China*.

**Table S1** Crystallographic Data of **TPE-TPY**

|  | **TPE-TPY** |
| --- | --- |
| empirical formula | C_41_H_29_N_3_ |
| fw | 563.67 |
| space group | *P21/c* |
| *a*, Å | 9.2740(3) |
| *b*, Å | 17.9959(5) |
| *c*, Å | 18.3236(4) |
| *β*, ° | 102.983(3) |
| *V*, Å^3^ | 2979.93(15) |
| *Z* | 4 |
| *ρ*_calcd_ g/cm^-3^ | 1.256 |
| *μ*, mm^−1^ | 0.074 |
| Radiation (*λ*, Å) | 0.71073 |
| temp, (K) | 143(10) |
| *R*1(*F*_o_)^a^ | 0.0784 |
| w*R*2(*F*_o_^2^)^b^ | 0.1996 |
| GOF | 1.059 |

^a^ *R*1 = Σ*F*_o_ - *F*_c_/Σ*F*_o_ ^b^*wR*2 = Σ[w(*F*_o_^2^ – *F*_c_^2^)^2^]/Σ[w(*F*_o_^2^)]^1/2^

**Figure S1** The synthetic routes of **1**


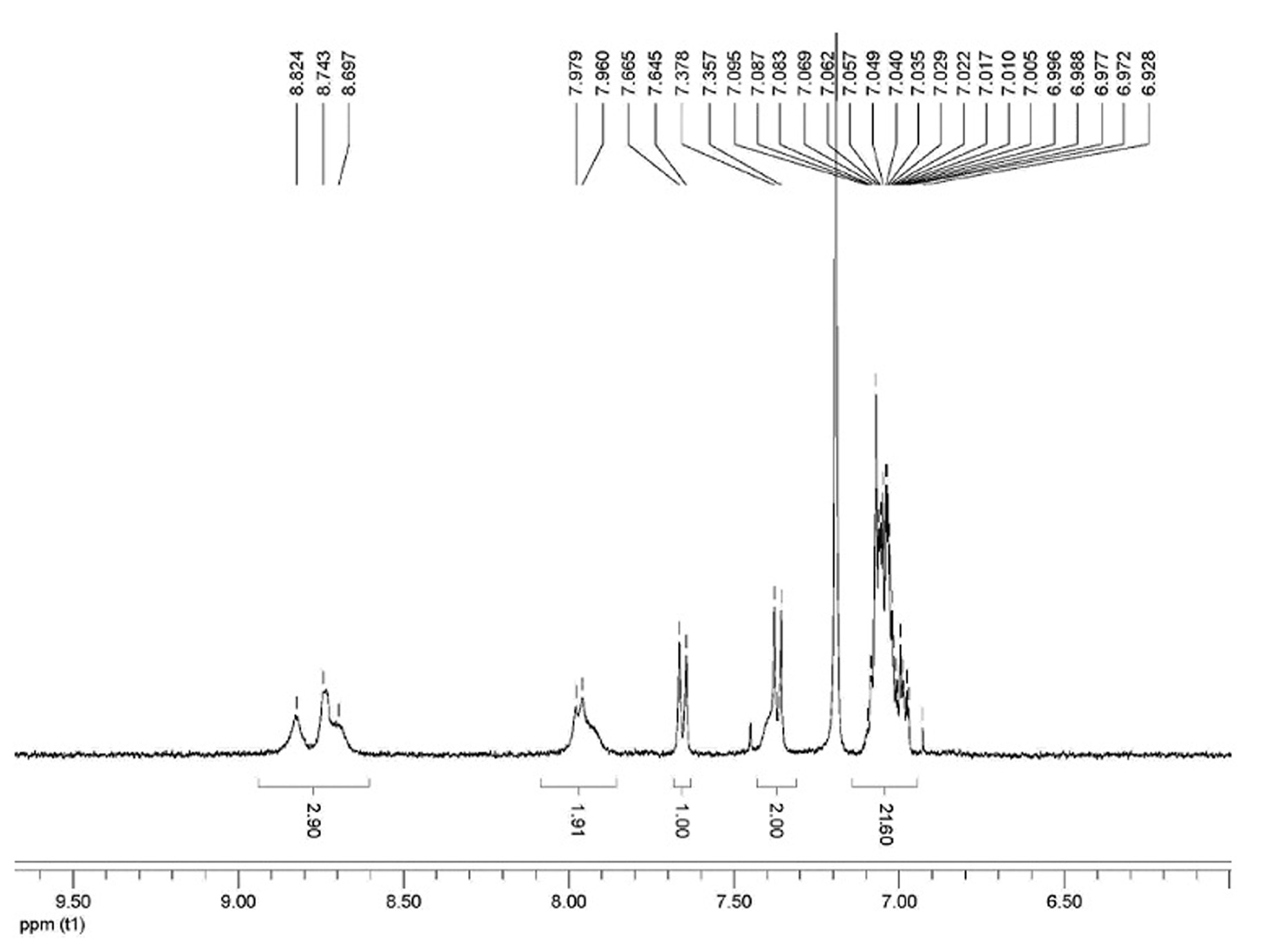


**Figure S2** ^1^H NMR spectra of **TPE-TPY** with 400 MHz in CDCl_3_ solutions

**TPE-TPY**: ^1^H NMR (400 MHz, CDCl_3_, TMS) δ (ppm): 8.78 (d, *J* = 32 Hz, 2H), 8.70 (s, 1H), 7.97 (d, *J* = 7.6 Hz, 2H), 7.65 (d, *J* = 8.0 Hz, 1H), 7.36 (d, *J* = 7.6 Hz, 2H), 6.93-7.10 (m, 22H).


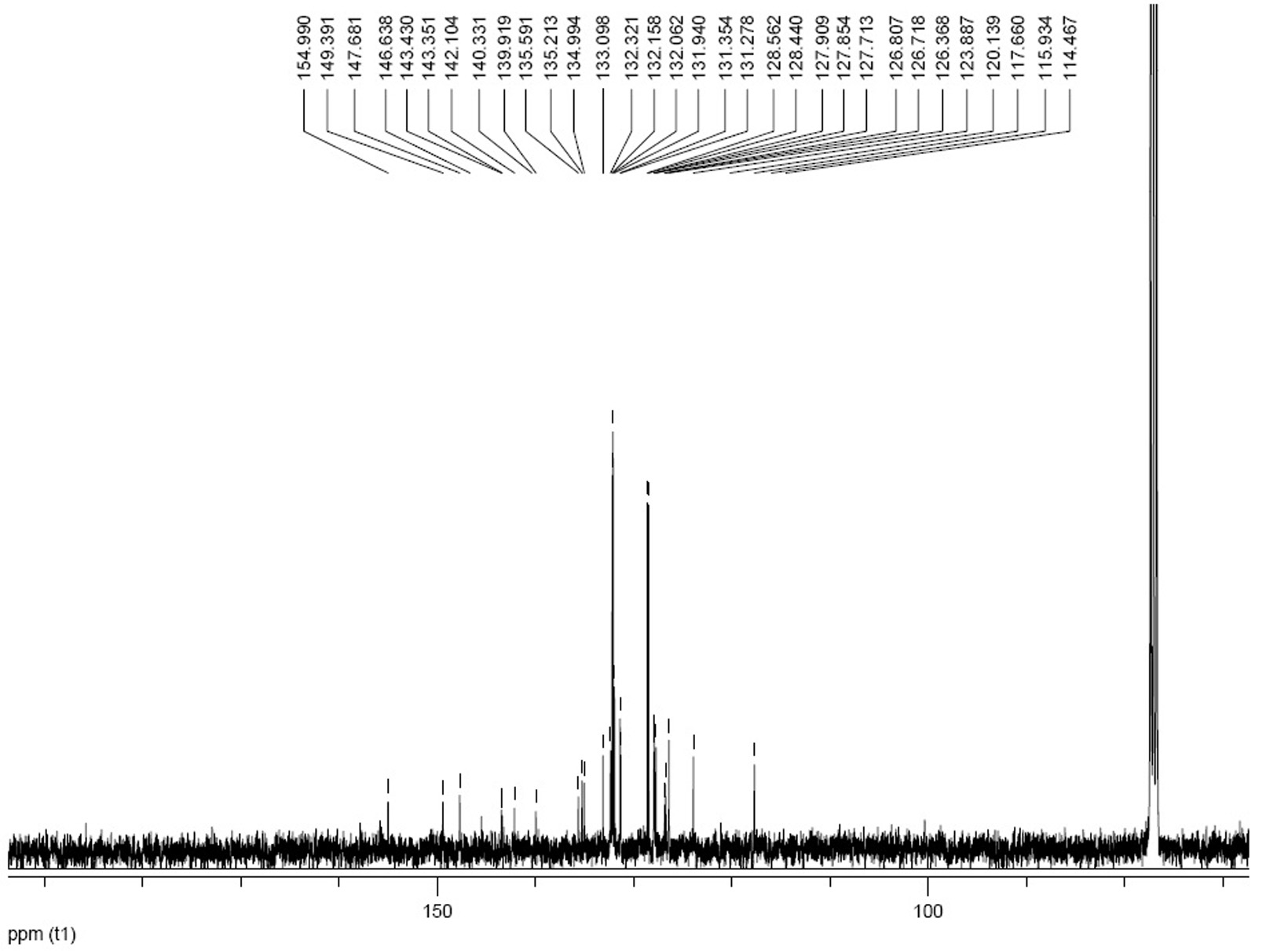


**Figure S3** ^13^C NMR spectra of **TPE-TPY** with 100 MHz in CDCl_3_ solutions

**TPE-TPY**: ^13^C NMR (100 MHz, CDCl_3_) δ (ppm): 155.0, 149.4, 147.8, 146.6, 143.4, 143.3, 142.1, 140.3, 139.9, 135.6, 135.2, 135.0, 133.1, 132.3, 132.0, 131.4, 131.3, 128.6, 128.4, 127.9, 127.8, 127.7, 126.8, 126.7, 126.4, 123.9, 120.1, 117.6, 116.0, 114.5.


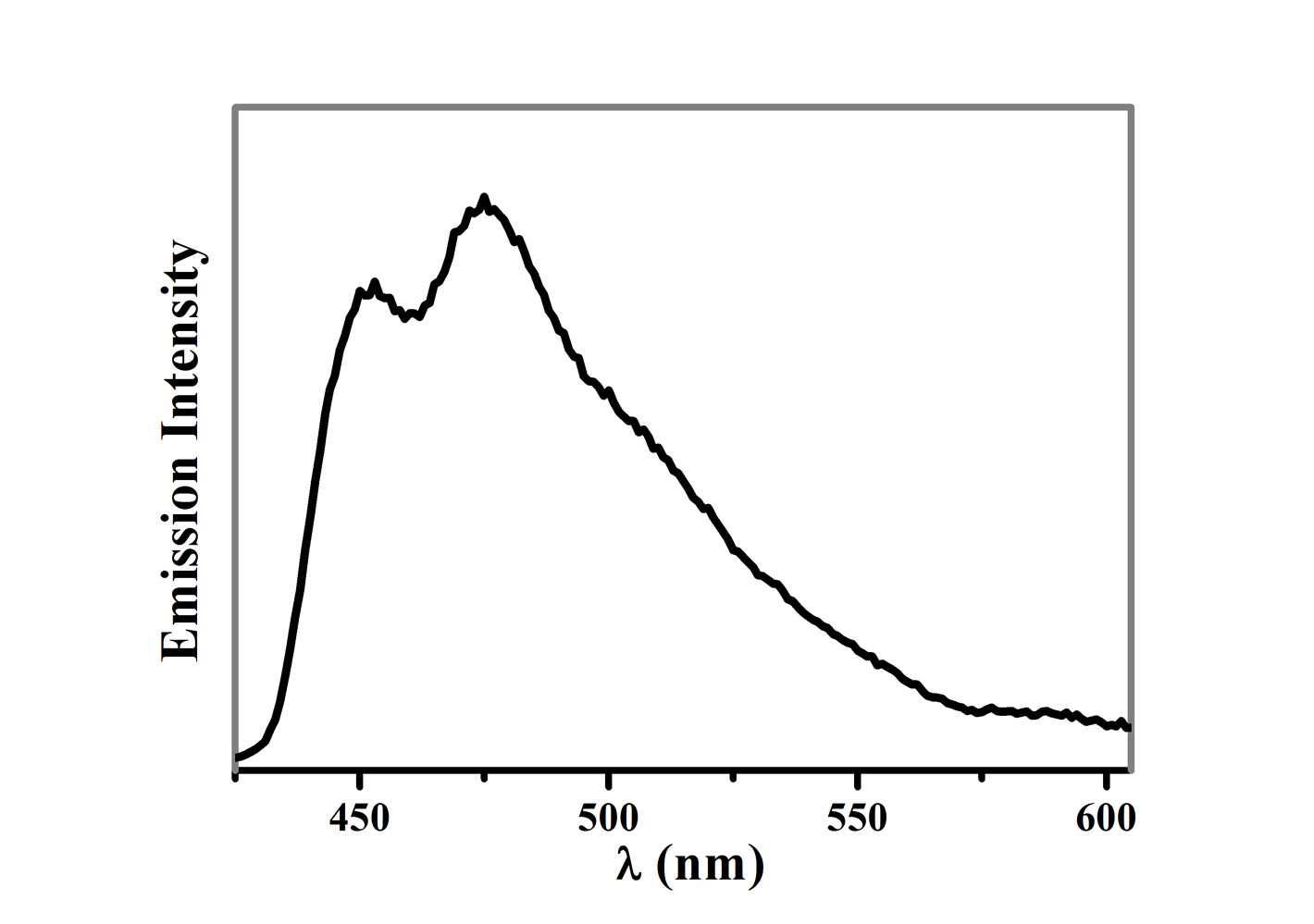


**Figure S4** Emission spectrum of **[TPE-TPY-Gd(hfac)_3_]** (*λ*_ex_ = 412 nm) in methanol at 77 K

**Figure S5** Emission spectra (*λ*_ex_ = 306 nm) of [**TPE-TPY-Eu(NO_3_)_3_]** and **[TPY-Eu(NO_3_)_3_]** in dichloromethane solutions (10^-5^ M), suggesting that intramolecular rotations of TPE consume the energies of the excited states of TPY, markedly reducing the efficiency of the energy transfer from TPY to Eu^III^ ion.

**Figure S6** Emission spectra (*λ*_ex_ = 380 nm) of **[TPE-TPY-Eu(NO_3_)_3_]** and **[TPY-Eu(NO_3_)_3_]** in different concentrations, suggesting that the **TPE**-**TPY** acts as the sole sensitizer for Eu^III^-based emission at higher concentration.

**Figure S7** Excitation (*λ*_ex_ = 440 nm, dash) and emission spectra (*λ*_ex_ = 386 nm, solid) of TPE-TPY in different concentration (inset is the excitation and emission spectra of TPE-TPY with the concentration from 10^-5^ M to 10^-2^ M).

**Figure S8** Excitation (*λ*_ex_ = 440 nm, dash) and emission spectra (*λ*_ex_ = 386 nm, solid) of TPE-TPY with different volume fractions of dichloromethane/*n*-hexane at ambient atmosphere.


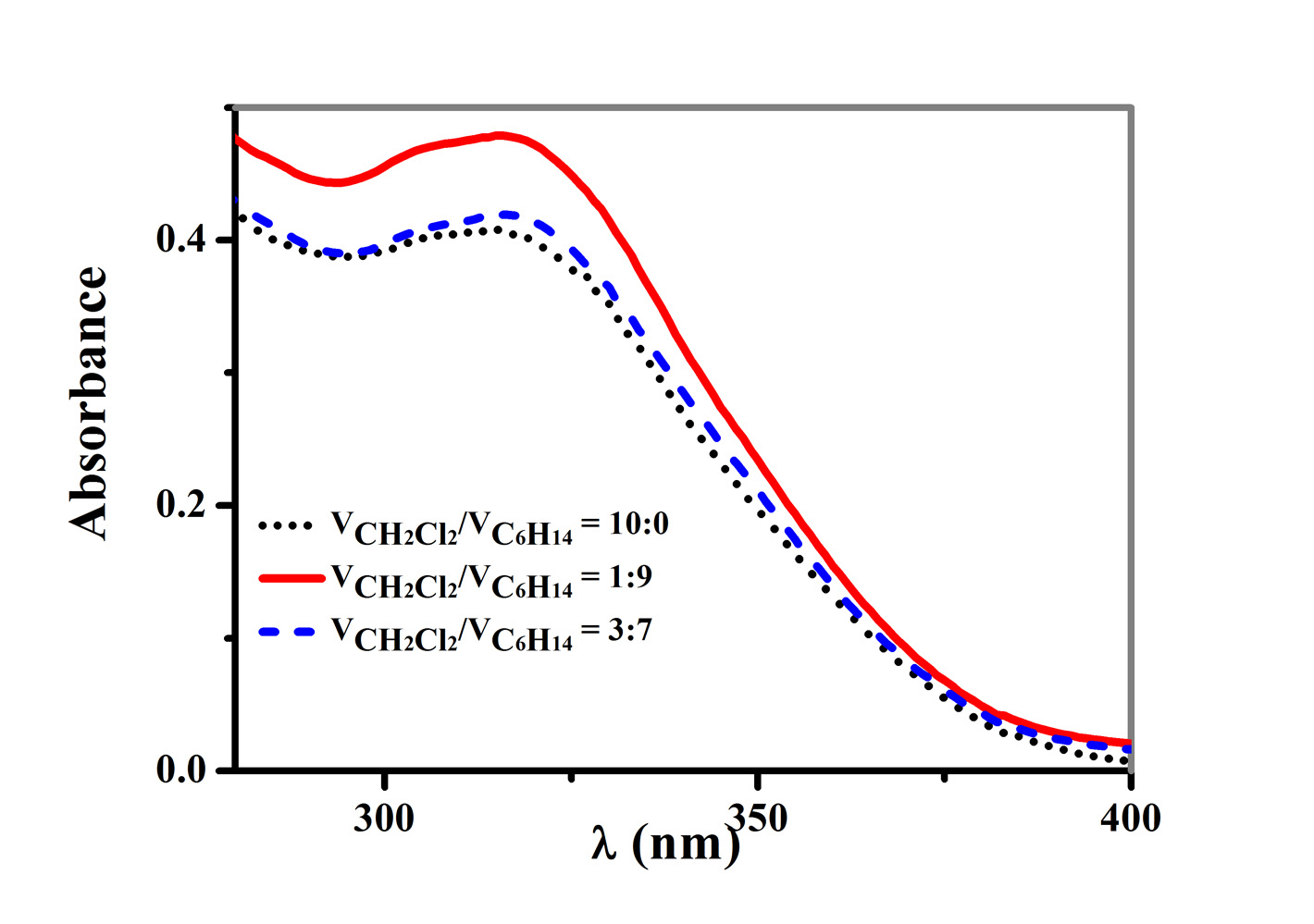


**Figure S9** Absorption spectra of **1** in dichloromethane/*n*-hexane mixtures, the volume fractions are 1:9, 3:7 and 10:0, respectively.


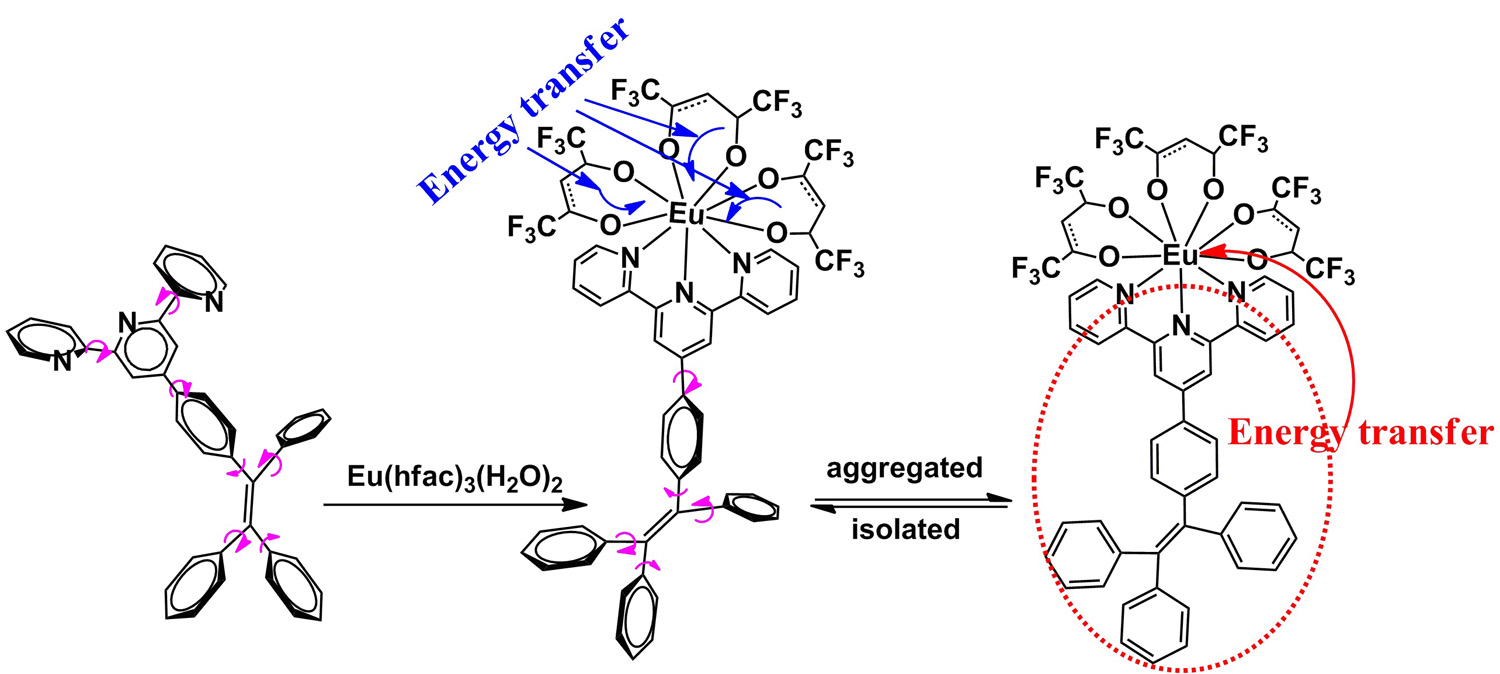


**Figure S10** Restriction of intramolecular rotation (**RIR**) process gradually results different energy transfer pathway in **1**.

**Figure S11** IR spectra of **1** IR (KBr, cm^-1^): 1655s (C=O), 1256s (C=C /C-CF_3_).


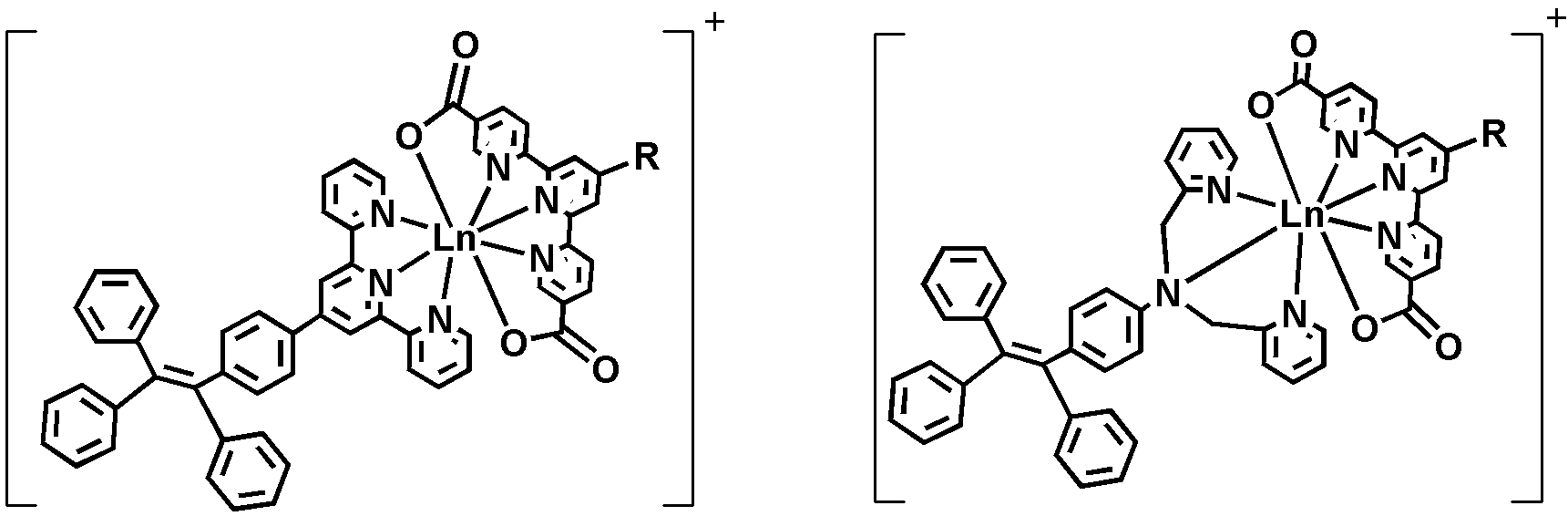


**Figure S12** Structures of ionic smart lanthanide bioprobes (R= recognize subunit)


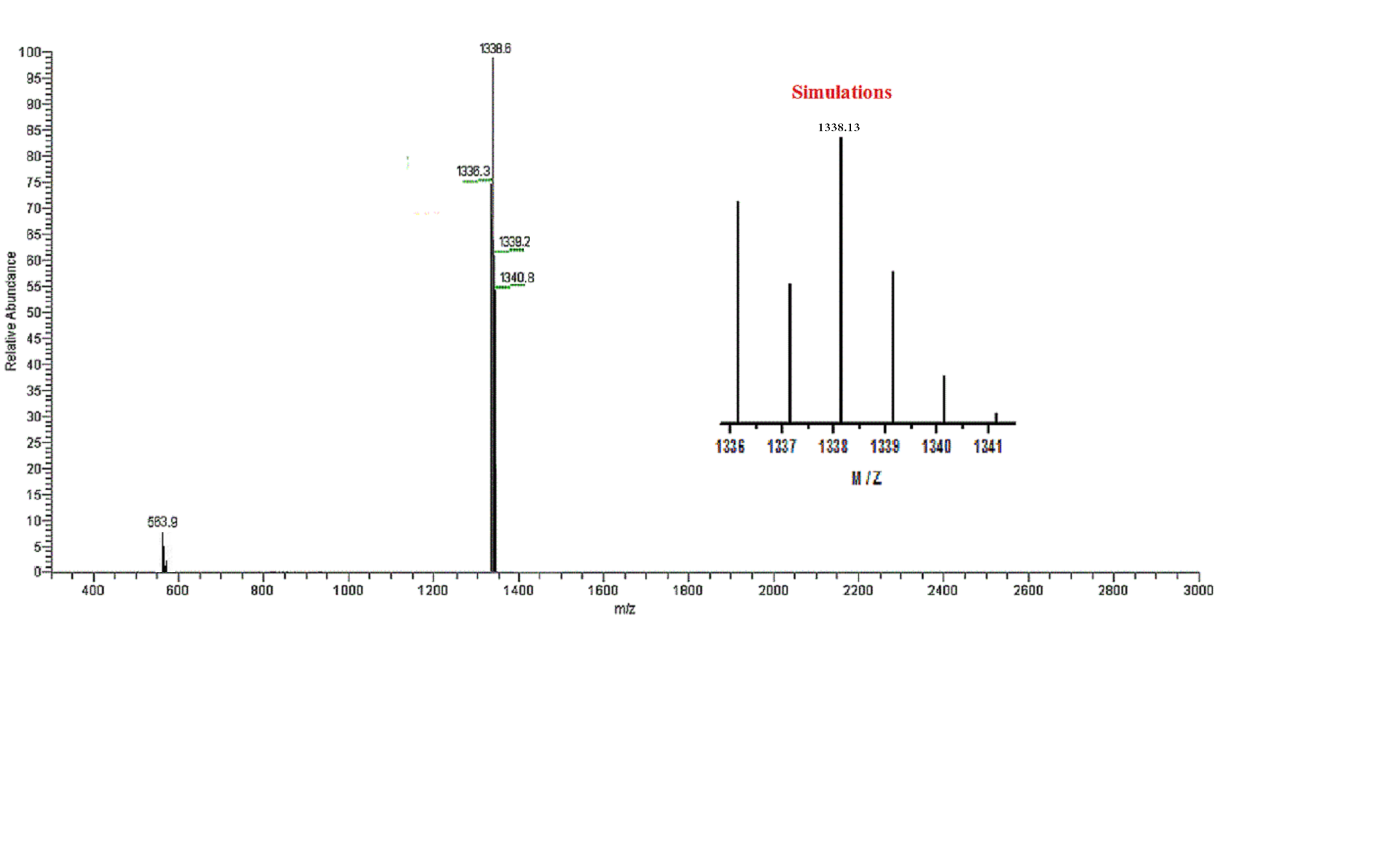
**Figure S13** Positive ion ESI-MS of **1**.

ESI-MS (CH_3_OH-CH_2_Cl_2_, *m/z*): [M+H]^+^, C_56_H_33_N_3_EuF_18_O_6_, M/Z: 1338.13 (100.0%); [TPE-TPY+H]^+^, C_41_H_30_N_3_, M/Z: 564.24 (7.0%).
